# A Human-specific Protein Regulated by Alternative Polyadenylation Shapes Uniqueness of Human Brain Development

**DOI:** 10.1093/gpbjnl/qzaf125

**Published:** 2025-12-13

**Authors:** Ting Li, Fan Mo, Jianhuan Qi, Chunqiong Li, Xiangshang Li, Jie Zhang, Yingfei Lu, Chao Yao, Li Zhang, Baoyang Hu, Chuan-Yun Li, Ni A An

**Affiliations:** State Key Laboratory of Protein and Plant Gene Research, Laboratory of Bioinformatics and Genomic Medicine, Institute of Molecular Medicine, College of Future Technology, Peking University, Beijing 100080, China; Institute of Genetics and Developmental Biology, Chinese Academy of Sciences, Beijing 100101, China; University of Chinese Academy of Sciences, Beijing 100049, China; University of Chinese Academy of Sciences, Beijing 100049, China; State Key Laboratory of Stem Cell and Reproductive Biology, Institute of Stem Cell and Regeneration, Institute of Zoology, Chinese Academy of Sciences, Beijing 100049, China; University of Chinese Academy of Sciences, Beijing 100049, China; State Key Laboratory of Stem Cell and Reproductive Biology, Institute of Stem Cell and Regeneration, Institute of Zoology, Chinese Academy of Sciences, Beijing 100049, China; State Key Laboratory of Protein and Plant Gene Research, Laboratory of Bioinformatics and Genomic Medicine, Institute of Molecular Medicine, College of Future Technology, Peking University, Beijing 100080, China; Institute of Genetics and Developmental Biology, Chinese Academy of Sciences, Beijing 100101, China; University of Chinese Academy of Sciences, Beijing 100049, China; State Key Laboratory of Protein and Plant Gene Research, Laboratory of Bioinformatics and Genomic Medicine, Institute of Molecular Medicine, College of Future Technology, Peking University, Beijing 100080, China; State Key Laboratory of Protein and Plant Gene Research, Laboratory of Bioinformatics and Genomic Medicine, Institute of Molecular Medicine, College of Future Technology, Peking University, Beijing 100080, China; Savaid Medical School, University of Chinese Academy of Sciences, Beijing 101408, China; State Key Laboratory of Protein and Plant Gene Research, Laboratory of Bioinformatics and Genomic Medicine, Institute of Molecular Medicine, College of Future Technology, Peking University, Beijing 100080, China; Institute of Genetics and Developmental Biology, Chinese Academy of Sciences, Beijing 100101, China; University of Chinese Academy of Sciences, Beijing 100049, China; Chinese Institute for Brain Research, Beijing 102206, China; University of Chinese Academy of Sciences, Beijing 100049, China; State Key Laboratory of Stem Cell and Reproductive Biology, Institute of Stem Cell and Regeneration, Institute of Zoology, Chinese Academy of Sciences, Beijing 100049, China; State Key Laboratory of Protein and Plant Gene Research, Laboratory of Bioinformatics and Genomic Medicine, Institute of Molecular Medicine, College of Future Technology, Peking University, Beijing 100080, China; Institute of Genetics and Developmental Biology, Chinese Academy of Sciences, Beijing 100101, China; University of Chinese Academy of Sciences, Beijing 100049, China; Chinese Institute for Brain Research, Beijing 102206, China; Southwest United Graduate School, Kunming 650092, China; Institute of Genetics and Developmental Biology, Chinese Academy of Sciences, Beijing 100101, China; University of Chinese Academy of Sciences, Beijing 100049, China

**Keywords:** Alternative polyadenylation, Human brain development, ORF-disrupting APA, *ZNF271P*, Human-specific protein

## Abstract

Although new genes and regulatory events have been linked to the uniqueness of human brain development, it is unknown whether alternative polyadenylation (APA) also contributes to shaping this key feature that differentiates humans from other species. Here, we present an atlas of APAs of the human brain and identified 161 development-related, open-reading-frame-disrupting APAs associated with the dynamic translation of protein products. Among the genes affected by these events, we identified *ZNF271P*, which encodes a human-specific protein when using the distal polyadenylation site, a site that preferentially occurs during early brain development. The cortical organoids derived from *ZNF271P*-knockout human embryonic stem cells seemed to exhibit accelerated development and maturation, resulting in a significant decrease in organoid size, implicating that *ZNF271P* is involved in features unique to human brain development. We thus highlight APAs as new regulators in shaping the unique aspects of human brain development.

## Graphical abstract

**Figure:**
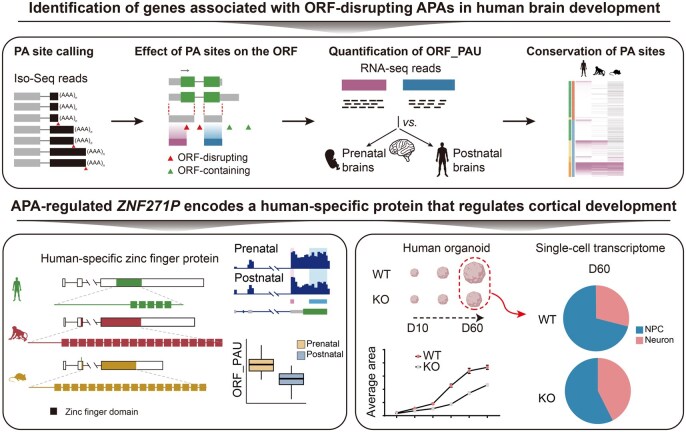

## Introduction

The brain has undergone significant expansion in size and complexity in humans since diverging from nonhuman primates and other mammals, and this expansion has contributed substantially to the higher levels of cognitive functions in humans [1–4]. Pilot comparative genomics studies have linked new human/hominoid-specific genes or regulatory events on existing genes to the uniqueness of human brain development [5–11], but whether other complex transcriptional regulations could also be involved in shaping this key feature that differentiates humans from other species is still not fully clarified.

Alternative polyadenylation (APA) is a co-transcriptional process in eukaryotes that generates messenger RNAs (mRNAs) and long non-coding RNAs with different 3′ ends through cleavage and polyadenylation [12–14]. The cleavage sites, also known as polyadenylation (PA) sites, can be located in the 3′ untranslated region (UTR), intronic region, the coding sequence (CDS), or even the 5′ UTR, according to classical gene annotations [15,16]. APA is involved in mRNA stability and subcellular localization, possibly because various regulatory elements may be present in the 3′ UTRs, such as microRNA (miRNA) binding sites and RNA-binding proteins (RBPs) [12,17–19]. Moreover, the use of a proximal PA site may lead to the disruption of the original open reading frame (ORF) and the production of truncated proteins or transcript isoforms without an in-frame stop codon [12,15,20]. It is thus interesting to investigate whether changes in this regulation may also participate in shaping uniqueness in human brain development.

Notably, previous studies have linked APA events in 3′ UTRs to temporospatial regulatory relationships in brain development and reported the tendency to use longer 3′ UTRs during neuronal differentiation [21–24]. Despite these efforts on APA regulation in the 3′ UTRs, relatively limited studies focus on APA events located within the 5′ UTRs, CDSs, or introns (referred to as internal APAs). A recent study highlights the roles of internal APAs in modulating protein levels during neural development [20]; nonetheless, a systematic survey of the internal APAs and their functions in human brain development, especially for the ORF-disrupting APA events, is urgently needed.

Recent advances in next-generation sequencing technology have led to the rapid growth of large-scale, systematic RNA sequencing (RNA-seq) data, facilitating the identification of APA events. However, short-read-based PA site identification is error-prone, especially for sites within repetitive regions [25–27]. Notably, Pacific Biosciences (PacBio) single-molecule isoform sequencing (Iso-Seq) can be used to sequence full-length polyadenylated mRNAs [28], providing an approach to accurately identify PA sites at the whole-transcript level [29]. It is thus practical to identify APA events and investigate their features and evolution with large-scale, short-read sequencing across multiple conditions, using the APA events identified by Iso-Seq as a reference to control for false positives.

At present, exploring the causal link between these novel regulatory events and human-specific traits remains challenging, as cell culture studies offer a restricted perspective on the advanced functions at the cellular or organ levels. Recent advances in human cortical organoid development can mimic early human brain development, thus providing a practical way to investigate this relationship [30–33]. More specifically, recent single-cell transcriptomic studies have suggested cross-species variation in cell types during primate brain development [34–36]; thus, it is intriguing to explore whether the candidate genes associated with species-specific PA regulation might play a role in cell type transitions, and consequently, the uniqueness of human brain development.

In this study, we generated an atlas of reference PA sites using Iso-Seq and performed a reference-based short-read sequencing study to identify genes associated with ORF-disrupting APAs involved in human brain development. Among these genes, we focused on *ZNF271P*, whose longer transcripts generated with distal PA sites encode a human-specific protein. Knockout of *ZNF271P* in human embryonic stem cells (hESCs) promotes the neuronal maturation of cortical organoids, indicating a potential role for this gene in increased brain size during human evolution.

## Results

### Identification of genes associated with ORF-disrupting APAs in human brain development

**Figure 1:**
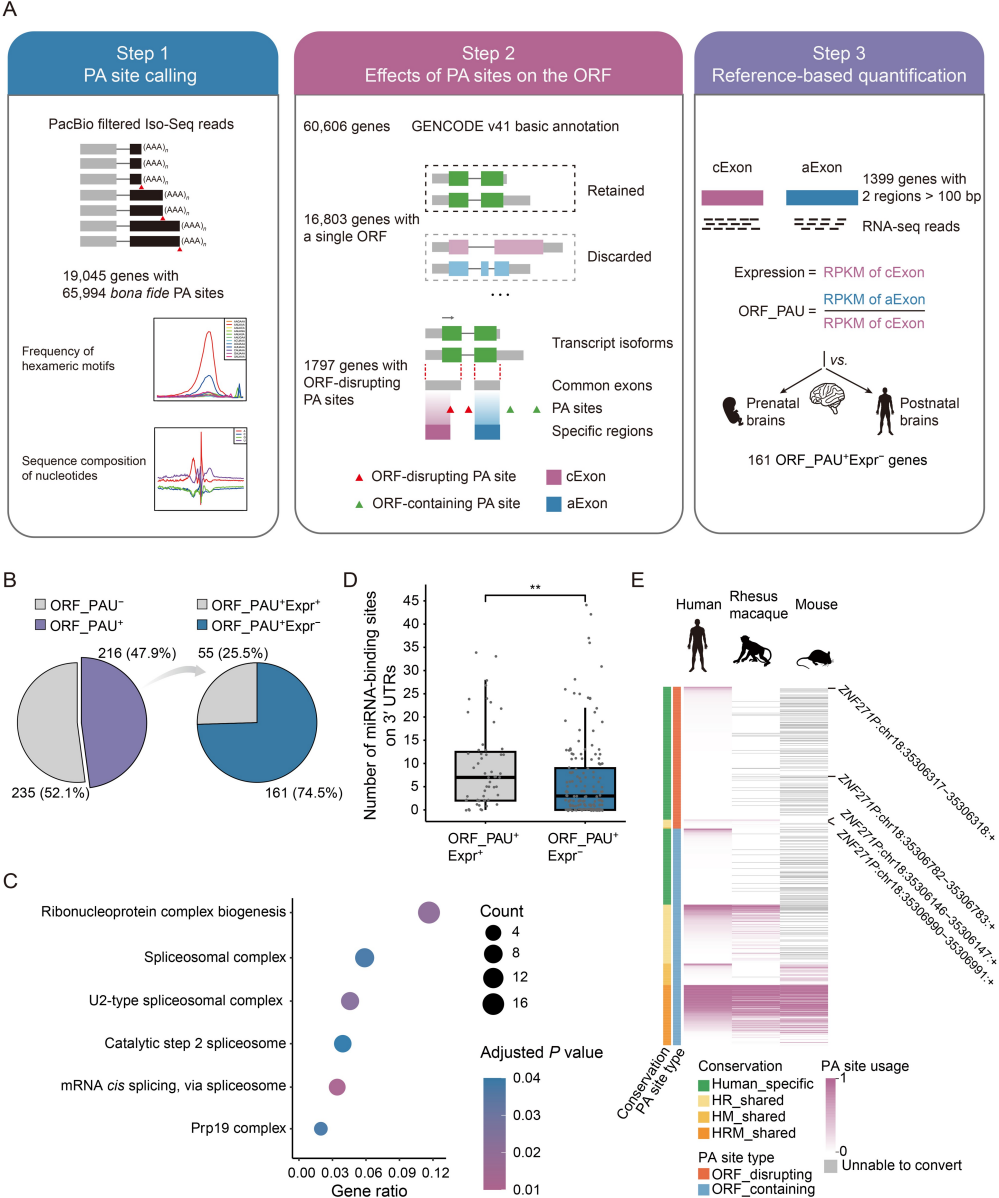
APA-directed ORF expression during human brain development and evolution **A**. Schematic diagram illustrating the identification and functional interrogation of PA sites in human brain development. Step 1: Identification and quantification of PA sites with Iso-Seq long-read sequencing. Red triangles: PA sites. Step 2: The effects of PA sites on the ORF. The exons of the transcript are shown in light gray boxes, with the coding regions highlighted in colored boxes. PA sites were classified into two categories: ORF-disrupting and ORF-containing, denoted by the red and green triangles, respectively. Purple boxes: the cExon shared by all isoforms; blue box: the aExon exclusive to isoforms harboring ORF-containing PA sites. Step 3: Quantification and comparison of the expression and ORF_PAU in prenatal and postnatal brains based on short-read sequencing. **B**. Pie chart showing the percentages of genes with (ORF_PAU^+^) or without (ORF_PAU^−^) significant changes in ORF_PAU between the prenatal and postnatal stages of human brain development (left panel). For the genes with significant changes in ORF_PAU, the percentages of genes with (ORF_PAU^+^Expr^+^) or without (ORF_PAU^+^Expr^−^) significantly changed expression are shown (right panel). **C**. Dot plot showing the Gene Ontology terms enriched among ORF_PAU^+^Expr^−^ genes, with the number of genes associated with each term and the significance of the enrichment shown. **D**. Boxplots showing the number of miRNA binding sites within the 3′ UTRs of ORF_PAU^+^Expr^+^ and ORF_PAU^+^Expr^−^ genes. *n* = 55 for ORF_PAU^+^Expr^+^ genes and *n* = 161 for ORF_PAU^+^ Expr^−^ genes. Wilcoxon test, **, *P* < 0.01. **E**. Heatmap showing the usage of PA sites in ORF_PAU^+^Expr^−^ genes in humans, as well as the usage of their orthologous sites in rhesus macaques and mice. The “PA site type” color bar indicates the type of PA sites: ORF-disrupting (orange) and ORF-containing (blue). The “Conservation” color bar indicates the conservation of the individual PA sites: Human_specific (green), detected exclusively in humans; HR_shared (light yellow), shared between humans and rhesus macaques; HM_shared (medium yellow), shared between humans and mice; HRM_shared (dark yellow), shared between humans, rhesus macaques, and mice. The color gradient in the heatmap represents PA site usage, with darker shades indicating higher usage. PA sites that could not be converted to rhesus macaques and/or mice are shown in gray for the respective species. Four ORF-disrupting PA sites on *ZNF271P* are labeled to emphasize disruptive usage and its conservation between humans and rhesus macaques. PA, polyadenylation; Iso-Seq, single-molecule isoform sequencing; ORF, open reading frame; cExon, constitutive exon; aExon, alternative exon; ORF_PAU, the usage of ORF-containing PA site; RPKM, the ratio of the reads per kilobase per million mapped reads; *vs*., *versus*; UTR, untranslated region.

To obtain an accurate list of APA events in human brain development, we first identified PA sites using 19 million full-length, non-chimeric reads generated from human brain Iso-Seq samples (see Materials and methods). A total of 65,994 PA sites in 19,045 genes were identified (**Figure 1**A; Table S1), which should represent *bona fide* PA sites. First, consistent with previous studies [37], we detected an enrichment of the canonical 3′ cleavage site motifs, such as AAUAAA, 50 base pairs (bp) upstream of these candidate PA sites, as well as canonical nucleotide sequence compositions surrounding the identified PA sites (Figure S1A and B). Notably, 74.3% of these PA sites were also identified by PolyASite (v2.0) [38] using a different strategy (3′ end sequencing to pinpoint candidate PA sites). The distribution of genomic locations of the identified PA sites is also consistent with previous reports (Figure S1C) [29].

For simplicity when studying the effects of PA site selection on the integrity of ORFs and subsequent protein abundance, we focused on 16,803 genes with a single ORF (Figure 1A; see Materials and methods). 5815 of these genes were covered with Iso-Seq reads, with a total of 17,686 PA sites identified (Table S2). In this list, 3717 PA sites in 1797 genes were identified as ORF-disrupting, located in the 5′ UTRs, CDSs, or intronic regions (Figure S1D; see Materials and methods). For each gene, we defined its constitutive exon (cExon) regions shared by all isoforms and alternative exon (aExon) regions shared by only ORF-containing isoforms, based on the positions of PA sites, and 1399 genes with minimal cExon and aExon lengths of at least 100 bp were then selected for further quantifications (Figure 1A; Table S3; see Materials and methods).

We then investigated whether these 1399 genes with ORF-disrupting PA sites could be involved in human brain development through the quantification and comparison of the usage of ORF-containing PA sites (ORF_PAU) and expression levels between prenatal and postnatal stages of human brains (Figure S2). To accurately quantify ORF_PAU, 451 genes with robust cExon expression were retained for further analysis. Of these, 216 genes with significant changes in ORF_PAU (|ΔORF_PAU| ≥ 0.15, adjusted *P* < 0.05) were identified between prenatal and postnatal stages of human brains (Figure 1B; Table S3; see Materials and methods), with 172 lengthening genes and 44 shortening genes in postnatal brains, indicating a preferential use of longer, ORF-containing PA sites in postnatal stages. As a note, 55 of the 216 genes were also differentially expressed between prenatal and postnatal stages of human brains (|log_2_ fold change| ≥ 1, adjusted *P* < 0.05; ORF_PAU^+^Expr^+^), while the remaining 161 genes exhibited changes only in ORF_PAU (ORF_PAU^+^Expr^−^, Figure 1B). The latter category of genes, which were merely regulated by APAs, were enriched for biological processes such as RNA splicing (Figure 1C), suggesting complex interactions between APA regulation and splicing [39,40]. Due to the relatively small number of ORF_PAU^+^Expr^+^ genes (55 genes), no significantly enriched pathway was identified in this category.

Interestingly, compared with genes in the ORF_PAU^+^Expr^+^ category, ORF_PAU^+^Expr^−^ genes showed significantly fewer miRNA binding sites in the 3′ UTRs (Figure 1D, Wilcoxon test, *P* = 3.1 × 10^−3^). No significant differences in either the expression levels or the 3′ UTR lengths between ORF_PAU^+^Expr^+^ genes and ORF_PAU^+^Expr^−^ genes were found (Figure S3). These findings thus indicated that the density of miRNA binding sites, rather than the length of the 3′ UTRs, might contribute to the differential expression of ORF_PAU^+^Expr^+^ genes between prenatal and postnatal stages of the human brain.

Interestingly, consistent with the evolutionary developmental biology hypothesis that proposes a shared mechanism between developmental processes and evolution [41], we found that most of these development-related PA regulatory changes were also species-specific. Briefly, for 161 ORF_PAU^+^Expr^−^ human genes, we identified 136 orthologous genes covered with Iso-Seq reads in both rhesus macaques and mice. Among these, the ORF-disrupting APA events (ORF_PAU < 1) were specifically found in humans for 107 genes (Table S4). To further explore the evolutionary context of individual PA sites, we identified orthologous ORF-disrupting PA sites in rhesus macaques and mice using Iso-Seq data from brain tissues (Materials and methods). For the majority of these ORF-disrupting PA sites (311 of 332, or 93.7%), the usage and the consequent disruption of the ORFs were detected exclusively in humans (Figure 1E; Table S5). As a positive control, we also examined the presence of orthologous ORF-containing PA sites in rhesus macaques and mice. Compared to ORF-disrupting PA sites, ORF-containing PA sites were significantly more conserved across species (Figure 1E, Fisher’s exact test, *P* < 2.2 × 10^−16^).

To control for false-positive findings due to sampling bias or sequencing discrepancies, we further downsampled the long-read counts from human and mouse samples to align with the sequencing depth in rhesus macaques. Accordingly, for 152 ORF-disrupting PA sites covered by sufficient reads after downsampling, 140 (92.1%) were detected exclusively in humans (Figure S4). Taken together, these species-specific APA events should contribute substantially to the flexible modulation of brain development during evolution.

### The longer isoform of *ZNF271P* encodes a human-specific protein

Prompted by the finding that some APAs may lead to the protein translation profiles biased toward specific developmental stages, we then subsequently identified *ZNF271P* for a proof-of-concept functional study among the 161 ORF_PAU^+^Expr^−^ genes (Figure 1E), considering its relatively higher usage of ORF-disrupting PA sites (Table S4), its stringent regulation during human brain development, and the fact that the zinc finger (ZNF) family of genes it belongs to play key roles in regulating proliferation, differentiation, and subsequent brain development [42–44].

**Figure 2:**
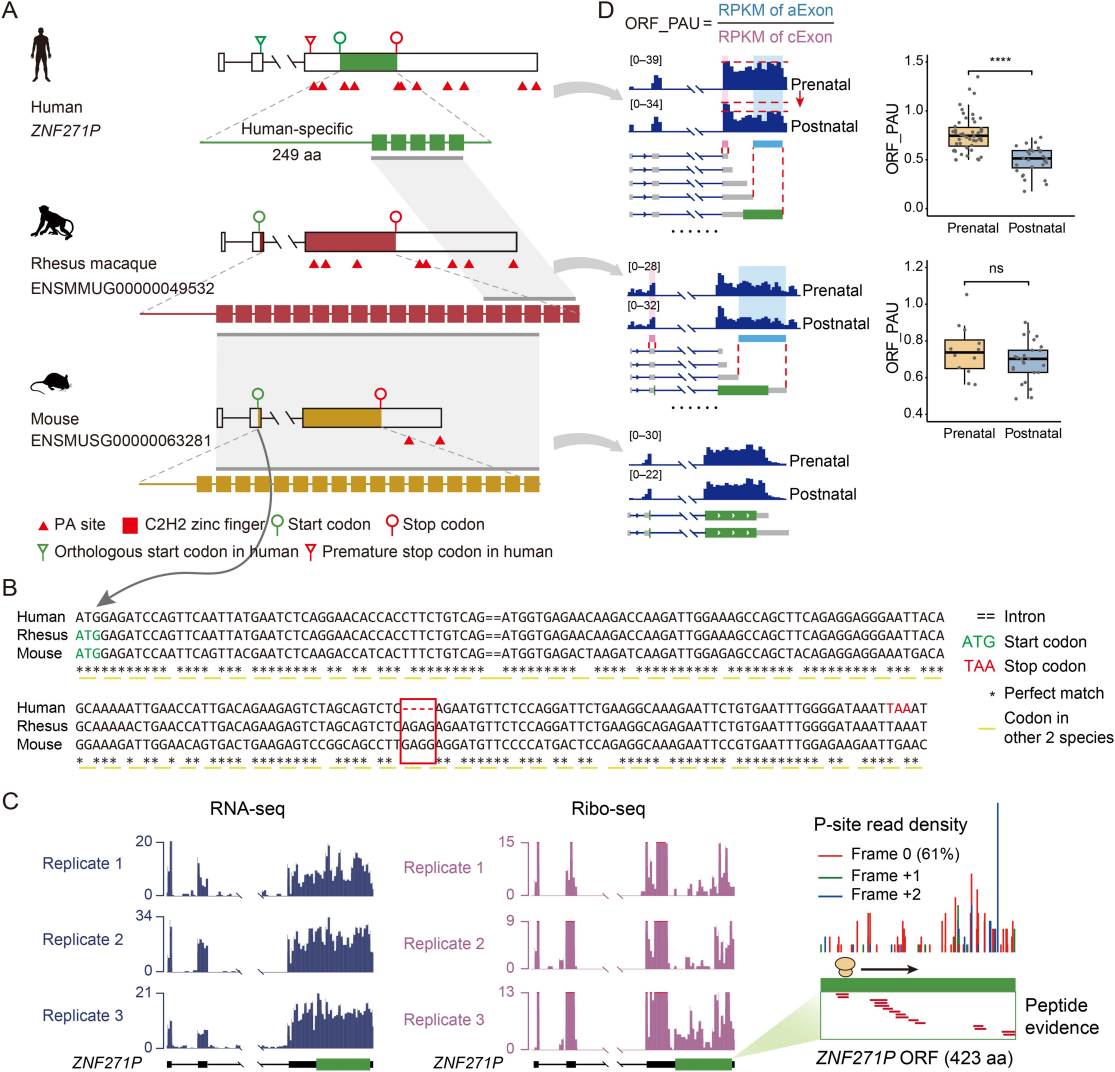
The evolution of *ZNF271P* **A**. Gene structures of *ZNF271P* in humans, rhesus macaques, and mice are shown, with the ORFs, PA sites, and protein domains highlighted at the corresponding coordinates. **B**. Cross-species alignment for a portion of the cDNA sequence of *ZNF271P* (from the start codon in rhesus macaques and mice to the premature stop codon of humans), with the 4-bp deletion leading to the premature stop codon in humans highlighted in red. **C**. Normalized coverage of RNA-seq reads from three biological replicates of human brains (left panel), and the corresponding normalized coverage of Ribo-seq reads (middle panel) at the genomic regions of *ZNF271P* are shown. The ORF regions are highlighted in thick green blocks. The ribosome profiling footprints at the ORF region and the peptides identified by high-throughput mass spectrometry aligned to the ORF region are shown (right panel). **D**. The left panel shows the normalized coverage of RNA-seq reads from prenatal and postnatal brains at the genomic regions of *ZNF271P* in three species. The sample sizes of prenatal/postnatal brains are as follows: humans (51/28), rhesus macaques (12/25), and mice (59/39). The transcripts with all ORF-disrupting PA sites and the first ORF-containing PA sites are aligned and shown below the coverage tracks. The cExon regions and aExon regions are indicated by the purple and blue shaded areas, respectively. The right panel displays boxplots showing the ORF_PAU of *ZNF271P* in prenatal and postnatal brains. Wilcoxon test, ****, *P* < 0.0001; ns, not significant. aa, amino acid; C2H2, Cys2His2; cExon, constitutive exon; aExon, alternative exon; RNA-seq, RNA sequencing; Ribo-seq, ribosome profiling sequencing.

Notably, during prenatal brain development, *ZNF271P* preferentially encodes longer isoforms with an ORF theoretically encoding a putative protein of 423 amino acids (aa). We then traced the evolutionary history of this new putative ORF in humans, rhesus macaques, and mice. Notably, the orthologs of *ZNF271P* in the other two species encode similar zinc-finger proteins, indicating that the ancestral locus is a protein-coding gene (**Figure 2**A). Examination of the *ZNF271P* orthologs in other species, including chimpanzees, dogs, and elephants, further verified the coding potential of the ancestral locus of *ZNF271P* (Figure S5; see Materials and methods). After the divergence between humans and chimpanzees, one human-specific 4-bp deletion emerged and interrupted the ancestral ORF, causing pseudogenization of the ancestral protein in humans (Figure 2B, Figure S6). However, when the start codon located at the last exon is used, the human *ZNF271P* locus seems to encode a new ORF of 423 aa. In the first part of the ORF, the 15 classic zinc finger domains in the ancestral proteins were removed due to the 4-bp deletion, leading to a 249-aa sequence specific to humans (Figure 2A, Figure S6). Moreover, one human-specific 1-bp insertion extended this ORF and turned the out-of-frame ORF into an in-frame ORF relative to the ancestral protein (Figure S6). These indels were verified to be fixed in humans by checking them in gnomAD v4, with the corresponding metadata of human samples presented in Table S6. The *ZNF271P* locus in humans thus encodes a chimeric ORF, with 249 aa specific to humans and the remaining 174 aa similar to the ancestral protein (Figure 2A).

The transcription and coding potential of this putative ORF was then verified by examining RNA-seq and ribosome profiling data from human brain samples (Figure 2C), and the periodicity of the footprints in ribosome profiling supported the translation of the ORF (proportions of frame 0, frame +1, and frame +2 footprints were 61%, 29%, and 10%, respectively). However, the ORF that used the ancestral ATG did not exhibit any protein-coding potential according to our ribosome profiling data. Also, the translation of the putative ORF was further supported by 15 distinct peptides (length > 20 aa) identified in previous mass spectrometry studies and archived in the PRoteomics IDEntifications database [45] (Figure 2C).

We then inspected the ORF_PAU of *ZNF271P* in human brains at different developmental stages. Notably, ORF_PAU (Figure 2D, Wilcoxon test, *P* = 9.8 × 10^−9^) and, subsequently, the expression of the ORF-containing isoforms in humans (Figure S7, Wilcoxon test, *P* = 8.0 × 10^−10^), were significantly higher in prenatal stages than in postnatal stages. The finding for the biased usage of the ORF-containing PA sites in prenatal stages was further verified using an independent dataset of transcriptomes from different developmental stages of the human brain (Figure S8). It is thus plausible that the isoform switching regulated by PA site selection may participate in the dynamic regulation of the protein level by fine-tuning the composition of different isoforms during brain development.

To further verify the regulation of APA events on *ZNF271P* expression and functions, we introduced clustered regularly interspaced short palindromic repeats (CRISPR)-associated protein 9 (Cas9) experiments to remove the ORF-containing PA sites of *ZNF271P* in human embryonic kidney 293T (HEK293T) cells, and then quantified the expression levels of the ORF-containing isoforms using reverse transcription-quantitative polymerase chain reaction (RT-qPCR) (Figure S9; Table S7; see Materials and methods). Notably, the expression level of the ORF-containing isoforms significantly decreased after knocking out the six ORF-containing PA sites (Student’s *t*-test, *P* = 8.8 × 10^−3^, Figure S9), verifying the direct regulation of APA on the expression level of *ZNF271P*-encoding isoform. The APA of *ZNF271P* during development is possibly regulated by RBPs. We identified 77 RBPs interacting with *ZNF271P*, supported by large-scale cross-linking and immunoprecipitation followed by next-generation sequencing (CLIP-seq) data based on the annotations in starBase (v2.0) [46] (Table S8). Among these candidates, the expression levels of two genes encoding RBPs, *ELAVL3* and *ELAVL1*, showed significant correlation with the ORF_PAU of *ZNF271P* across different developmental stages (Spearman’s rank correlation test, adjusted *P* = 1.6 × 10^−3^ and 3.6 × 10^−4^, estimates = 0.41 and 0.46, respectively). According to the CLIP-seq profiles, both *ELAVL3* and *ELAVL1* were predicted to bind the sequences within a 100 bp range of the PA sites of *ZNF271P* (Figure S10). In line with these findings, the ELAV family has been reported to play vital roles in alternative polyadenylation and splicing in nervous systems [47,48].

Taken together, these findings indicate that the ancestral ORF of the *ZNF271P* locus was disrupted by human-specific mutations, while a newly originated ORF encodes a human-specific protein in this region, with a biased expression profile in the early stage of brain development (Figure S7), shaped by a previously existing APA event conserved between humans and rhesus macaques. This newly originated human-specific protein may thus contribute to the uniqueness of early brain development in humans.

### *ZNF271P* regulates human cortical development

**Figure 3:**
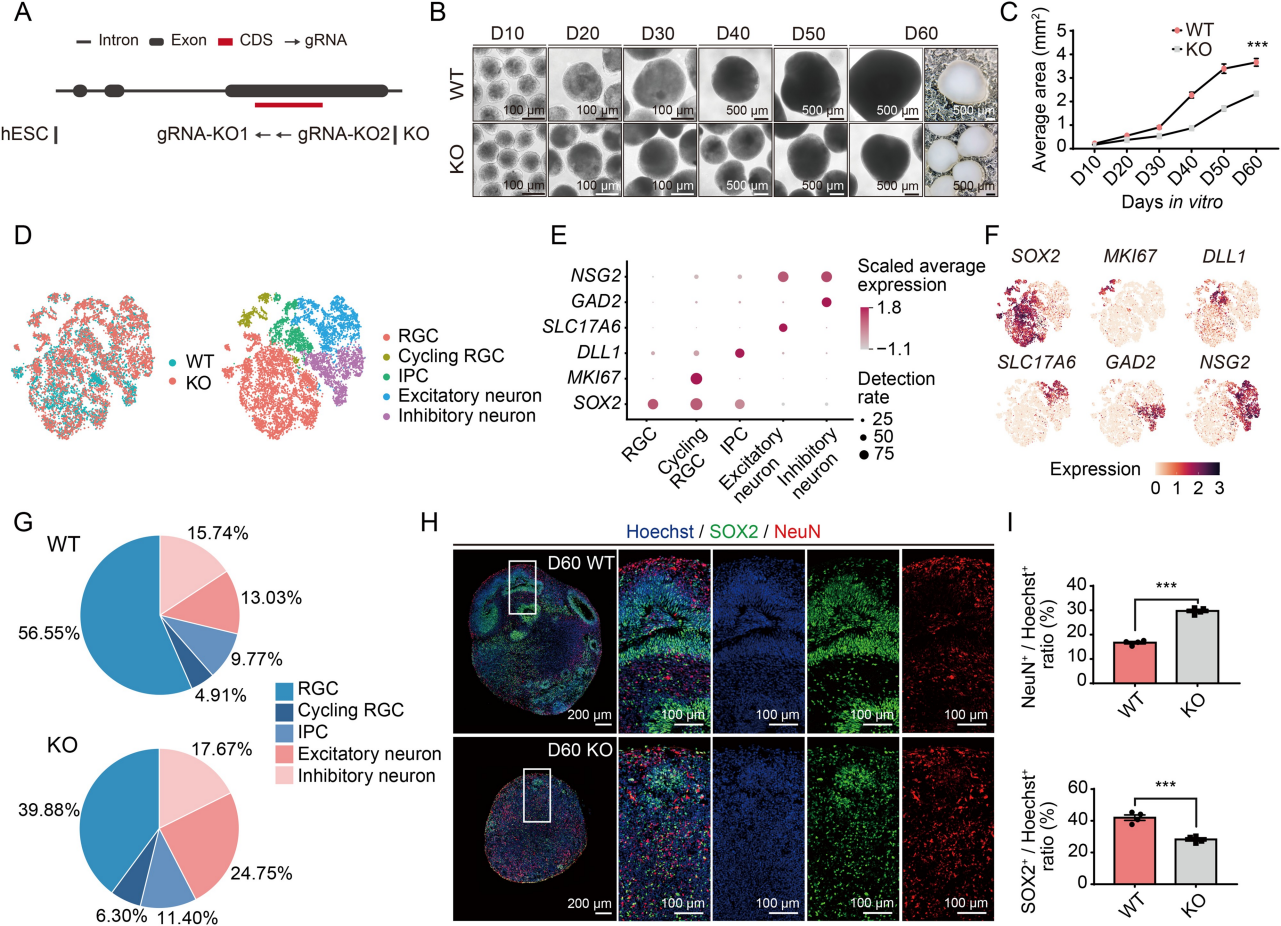
*ZNF271P* regulates neuron maturation **A**. Overview of CRISPR/Cas9-mediated gene-editing in hESCs, in which the gRNAs were designed to target the CDS regions (KO) of *ZNF271P*. **B**. Brightfield images showing the sizes of organoids generated from wild-type hESCs (WT) and *ZNF271P*-knockout hESCs (KO). D10–D60: protocol days 10, 20, 30, 40, 50, and 60. Scale bars from D10 to D30, 100 μm. Scale bars from D40 to D60, 500 μm. **C**. Quantification of the average area of organoids generated from wild-type hESCs (WT, red points) and *ZNF271P*-knockout hESCs (KO, gray points), according to brightfield images, *n* = 30 for each group and each time point. ***, *P* < 0.001 (Student’s *t*-test). **D**. Single cells from organoids grown for 60 days represented in t-SNE space. Cells on the left and right of the t-SNE plot are color-coded by sample and cell type, respectively (WT, organoids generated from wild-type hESCs; KO, organoids generated from *ZNF271P*-knockout hESCs). **E**. Dot plot showing the distribution of marker gene expression in different types of cells as defined by single-cell transcriptome sequencing. **F**. Feature plots showing the expression of marker genes indicative of several major cell populations (*SOX2^+^MKI67^−^DLL1*^−^, RGCs; *MKI67*, dividing RGCs; *DLL1*, IPCs; *SLC17A6*, excitatory neurons; *GAD2*, inhibitory neurons; *NSG2*, neurons). **G**. Pie charts showing the proportions of different types of cells in organoids grown for 60 days from wild-type hESCs (WT, upper panel) and *ZNF271P*-knockout hESCs (KO, lower panel). **H**. Immunofluorescence staining of SOX2 (green) and NeuN (red) in Hoechst-stained (blue) organoids grown for 60 days generated from wild-type hESCs (WT) and *ZNF271P*-knockout hESCs (KO). Scale bars, 100 μm (first column) and 200 μm (columns 2–5). **I**. Quantification of immunofluorescence staining. *n* = 4 organoids. ***, *P* < 0.001 (Student’s *t*-test). CRISPR/Cas9, clustered regularly interspaced short palindromic repeats-associated protein 9; gRNA, guide RNA; hESCs, human embryonic stem cells; CDS, coding sequence; t-SNE, t-distributed stochastic neighbor embedding; WT, organoids generated from wild-type hESCs; KO, organoids generated from *ZNF271P*-knockout hESCs; RGC, radial glial cell; IPC, intermediate progenitor cell.

To further clarify the function of this human-specific gene in neurogenesis and brain development, we then produced hESCs with CRISPR/Cas9-induced *ZNF271P* knockout and investigated the effect of *ZNF271P* depletion on the development of human cortical organoids (**Figure 3**A, Figure S11; see Materials and methods). Although the depletion of this gene through the partial deletion of its third exon did not significantly affect the pluripotency of hESCs (Figure S12), the organoids grown from *ZNF271P*-knockout hESCs (*ZNF271P*-KO) exhibited a significant reduction in size compared to wild-type organoids at the corresponding developmental stages (Figure 3B and C, Student’s *t*-test, *P* = 6.1 × 10^−10^).

We subsequently conducted a single-cell transcriptome analysis to determine whether alterations in organoid cell composition might account for the observed reduction in organoid size. Considering the mature organoids at culture day 60 (D60) recapitulated more cell types and neuronal functions, we thus chose D60, which also showed the most significant size differences in our data, for further study. Overall, the transcriptomes of 13,720 single cells from organoids grown for 60 days from wild-type or *ZNF271P*-KO cells were profiled (Figure 3D–F; see Materials and methods). Although major cell types such as radial glial cells (RGCs), intermediate progenitor cells (IPCs), and excitatory or inhibitory neurons could be detected in both samples, the organoids grown from *ZNF271P*-KO cells showed a higher proportion of neurons and a lower level of neural progenitor cells (NPCs) (including RGCs, cycling RGCs, and IPCs) than those grown from wild-type cells (Figure 3G). To reconstruct the cell differentiation trajectories, we performed pseudotime analysis for the development of the organoids (D60, Figure S13). The pseudotime analysis elucidated the progression from neural progenitor cells to neurons (Figure S13A). To compare the differentiation states between organoids grown from wild-type and *ZNF271P*-KO cells, we quantified the proportion of cells at various pseudotime differentiation stages and found a higher proportion at the late stages of differentiation in organoids grown from *ZNF271P*-KO cells (Figure S13B). We further verified the findings of the single-cell transcriptome study and clarified the changes in the composition of progenitor cells and neurons using immunofluorescence staining with cell type-specific markers (Figure 3H and I). Consistently, a marked reduction in the ratio of SOX2^+^ NPCs was observed in organoids grown from *ZNF271P*-KO cells (Figure 3H and I, Student’s *t*-test, *P* value = 4.7 × 10^−4^), contrasting with the elevated ratio of NeuN^+^ cells (mature neuronal populations) (Figure 3H and I, Student’s *t*-test, *P* = 1.4 × 10^−2^). These findings indicate that the organoids derived from *ZNF271P*-KO cells exhibit accelerated neuronal maturation.

To further investigate the functions of *ZNF271P*, we analyzed the genes differentially expressed between NPCs of organoids derived from *ZNF271P*-KO and *ZNF271P*-WT cells and identified 75 differentially expressed genes (Table S9). These genes were enriched in functional terms such as the regulation of neurogenesis (hypergeometric test, adjusted *P* = 2.8 × 10^−4^) (Table S9), a pathway possibly underlying the phenotypic changes observed in the *ZNF271P*-KO experiments.

In conclusion, the organoids derived from *ZNF271P*-KO cells seemed to exhibit accelerated development and maturation, resulting in a significant decrease in the organoid size at the same developmental stage. Since the CRISPR/Cas9 gene editing system showed no off-target effects, it is unlikely that the phenotypes in *ZNF271P*-KO organoids resulted from off-target effects (Table S10; see Materials and methods). It is thus plausible that this human-specific protein could contribute to the evolution of an extended period of brain development and subsequently the enlargement of the human brain through the regulation of NPC expansion and neurogenesis.

## Discussion

APA regulation is an important mechanism in mammals that generates transcripts with varied lengths. According to the Iso-Seq data used in this study, 10,657 of 14,464 protein-coding genes and 1820 of 4531 non-coding genes were polyadenylated at more than one site in human brains, indicating the prevalence of this regulation in modulating isoform switching and transcript composition for both protein-coding and non-coding genes. Briefly, APA-directed isoform switching fine-tunes neurodevelopmental genes in two ways: (1) APA alters the 3′ UTR length, affecting miRNA and RBP binding sites, thus influencing protein synthesis and localization [24,49]; and (2) APA disrupts the ORF, modulating protein abundance [20]. Our study focused on the latter, where APA regulates protein levels during development. In contrast to the widespread regulation by APA and the large number of APAs associated with the transcript life cycle, we identified a limited number of genes (*n* = 216) regulated by ORF-disrupting APA during brain development, likely controlled by canonical polyadenylation factors and RBPs, with additional mechanisms to be explored [50,51]. Additionally, functional experiments on the human-specific protein regulated by developmental APA, *ZNF271P*, further highlighted the roles of APA regulation in shaping brain uniqueness.

In comparison to APAs that fine-tune the stability and subcellular localizations of transcripts, APA events associated with dynamic translation of protein products are more likely to have been removed by purifying selection due to their more deleterious effects on cellular homeostasis. As expected, the distribution of ORF_PAU for the majority of the 1399 genes (or 61.6%) was within the 0.9–1 range (Figure S14). However, we also observed that some APA events associated with truncated protein products exhibited relatively higher usage. This may be due to their presence in human populations by chance or because they are maintained for their potential adaptive functions, particularly in the regulation of human development through isoform switching.

In this study, we identified development-related ORF-disrupting APA events by comparing RNA-seq data from prenatal and postnatal brain samples. To ensure that our findings were not confounded by the potential batch effects caused by the gender and age differences in these samples, we implemented a multilevel analysis. First, upon comparing ORF_PAU across different gender groups, we observed no significant differences (26 females *vs.* 53 males; Wilcoxon test, *P* > 0.05). Furthermore, the ComBat function of the sva (v3.50.0) package was used to remove the potential batch effect caused by gender (Figure S15A). After removing the batch effects, we identified 201 ORF-disrupting, development-related APA events. Notably, all 201 events are included in the previously identified 216 events. Second, we performed hierarchical clustering analysis to assess the similarity of the ORF_PAU profiles at subdivided age or time points (embryonic: 4–7 weeks; early fetal: 8–12 weeks; early mid-fetal: 13,16,18 weeks; late mid-fetal: 19 weeks), for genes identified as shortening or lengthening between prenatal and postnatal stages. We found that the ORF_PAU patterns across subdivided age/time points at prenatal and postnatal stages clustered together, indicating the consistency across different developmental stages (Figure S15B).

The global pattern we observed, where more genes utilize longer isoforms in postnatal brains compared to prenatal stages, aligns with findings from previous studies [52–54]. This trend is likely regulated by the temporospatial expression of key factors of canonical cleavage and polyadenylation [54–57]. For example, the increased expression of canonical cleavage factors during early development promotes the use of proximal PA sites. However, prenatal brain development, which involves neuron proliferation, differentiation, and migration, is a dynamic and complex process [58]. Thus, other complex regulatory mechanisms, such as RBPs and post-transcriptional modifications, may also contribute to this process. Consequently, some genes showed a preference for shorter isoforms in postnatal stages, including *ZNF271P*. Taken together, while the overall trend demonstrated a preference for longer, ORF-containing isoforms in postnatal brains, *ZNF271P* was selected for further investigation due to its unique evolutionary path, expression profile, and potential functional significance.

The human-specific protein ZNF271P was preferentially expressed during early brain development (Figure S7), with its expression shaped by a conserved APA regulatory event shared between humans and rhesus macaques. ZNF271P protein belongs to the Cys2His2 (C2H2) ZNF gene family, typically characterized by an average of 10 finger motifs [59,60]. However, the human ZNF271P protein exhibited a reduced number of zinc fingers compared to its orthologs, likely due to degeneration processes (Figure 2A). This specific loss may affect its binding specificity and function. Consistently, mCherry-tagged ZNF271P transfection in HEK293T cells revealed cytoplasmic localization (Figure S16), suggesting its function has diverged from the ancestral zinc finger protein.

Although knockout experiments could also affect gene regulation, the removal of the ZNF271P protein is likely the primary driver of the observed phenotype. First, the 41-bp deletion did not overlap with key regulatory elements, such as miRNA binding sites or candidate *cis*-regulatory elements. Second, a rescue construct integrating only the CDS of *ZNF271P* into the adeno-associated virus integration site 1 locus in KO cells partially rescued the phenotype (Figure S17). Brain organoids grown from these rescue cells exhibited increased size, fewer neurons, and more radial glial cells compared to organoids from *ZNF271P*-KO, highlighting the protein’s primary contribution. Notably, the incomplete rescue likely resulted from lower expression levels (∼ 12% of WT), possibly due to incomplete transcriptional regulation, post-transcriptional modifications, or unresolved epigenetic changes. While this study focuses on *ZNF271P*’s role in brain development, we also observed its ORF-containing isoforms expressed across multiple human organs, with higher levels in the kidney and ovary (Figure S18). These findings suggest *ZNF271P* may play broader roles in other tissues, which remains to be explored in further studies.

This study provides valuable insights but also has limitations for future exploration. First, due to the cost constraints of Iso-Seq, we relied on identified PA sites as a reference for quantifying isoform dynamics using RNA-seq. Advances in sequencing technology and cost reduction will enable a more precise investigation of APA dynamics at the single-site level. Second, while we identified potential RBPs regulating *ZNF271P* isoform switching during development, further experimental validation is needed, and other regulatory mechanisms remain to be explored. Third, knockout experiments indicate that ZNF271P protein depletion is the main factor responsible for the observed phenotypic changes in human organoids. However, additional studies, such as precise start codon knockouts, are required to exclude potential confounding effects on RNA isoforms. Finally, the molecular mechanisms linking this human-specific protein to NPC expansion, neurogenesis, delayed brain development, and subsequently brain enlargement warrant further investigation.

## Materials and methods

### Identification of PA sites with Iso-Seq

The processed Iso-Seq datasets for nine human brain samples were downloaded from the Encyclopedia of DNA Elements (ENCODE) database (Table S11). Full-length non-chimeric reads were mapped to the human reference genome (hg38), with errors corrected by TranscriptClean (v2.0.2). To further eliminate potential false positives due to internal priming, alignments containing stretches of adenosines were removed as described previously [29]. Finally, the clean Iso-Seq reads were then pooled together for the identification of candidate PA sites with a pipeline we described previously [61].

PA sites identified by PolyASite (2.0) [38] were obtained from https://polyasite.unibas.ch/download/atlas/2.0/GRCh38.96/atlas.clusters.2.0.GRCh38.96.bed.gz. We then compared the PA sites identified by Iso-Seq data with those identified by PolyASite (2.0), with all of these candidate PA sites extended 15 bp from the center for the comparison of the positional overlaps using BEDTools (v2.26.0) [62]. movAPA (v0.2.2) was used to annotate the identified PA sites onto their respective genomic coordinates, including 3′ UTRs, 5′ UTRs, CDSs, exons, intergenic regions, and introns.

### Quantification of gene expression and ORF_PAU during human brain development

For simplicity, when studying the effects of PA site selection on the integrity of ORFs and subsequent protein abundance, we focused on PA sites situated within genes with a single ORF. For protein-coding genes, the coordinates for coding sequences were obtained from the GENCODE (v41) database. For genes not annotated as “protein-coding”, such as long non-coding RNAs or pseudogenes, the sequences of the transcripts were downloaded from Ensembl (v107), and the putative ORFs were predicted with EMBOSS (v6.6.0.0) [63]. For each transcript, the longest ORF (with a threshold of the length ≥ 100 aa) was retained. All the PA sites were divided into two groups (ORF-disrupting and ORF-containing) based on their position relative to the end of the ORF. For each gene with ORF-disrupting PA sites, the cExon common to all isoforms and the aExon specific to isoforms containing the ORF were delineated based on the positional context of the PA sites. To eliminate the confounding effect of genomic overlap with other genes on the quantification of PA usage, we precisely excised the overlapped region from both the cExon and aExon regions. We excluded genes with cExon or aExon lengths less than 100 bp to ensure accurate quantification of PA usage.

RNA-seq data from developing human brains were generated in a previous study [64] and downloaded for use here. In summary, this dataset included 51 prenatal brain samples across gestational ages ranging from 4 to 19 weeks and 28 postnatal samples spanned a broad developmental spectrum from neonatal stages (day 0 post-birth) to elderly individuals (up to 58 years old) (Table S12). The binary alignment map (BAM) files were converted to FASTQ files and remapped to the human reference genome (hg38) using HISAT2 (v2.2.1) [65], and only uniquely mapped reads were kept. Only samples with adequate fragmentation (fragmentation score > 0.885) in RNA-seq were kept in the following analyses to exclude the effects of 3′ sequencing bias on PA identification [66].

Prenatal and postnatal samples were compared to identify the expression levels and changes in ORF_PAU associated with development. In brief, we estimated the expression of cExons and aExons with featureCounts (v2.0.2) [67], and the ratio of the reads per kilobase per million mapped reads (RPKM) of the aExon to that of the cExon was then calculated to estimate ORF_PAU, assuming that the expression of the cExon was representative of the expression of the gene. Only genes with robust cExon expression (RPKM ≥ 1 in all samples) were retained for downstream analyses. Wilcoxon tests were performed to assess whether the expression or ORF_PAU of the genes significantly differed between the two groups, using a Benjamini–Hochberg corrected *P* < 0.05, with a threshold of either a fold change in expression ≥ 1 or a difference in ORF_PAU ≥ 0.15. Gene Ontology analyses were then performed with clusterProfiler (v4.4.4) [68] to identify function terms that were enriched in gene sets with an adjusted *P* value threshold of 0.05.

To further validate the difference in ORF_PAU between prenatal and postnatal stages of *ZNF271P*, we incorporated an independent dataset from Duffy and colleagues [69]. This dataset included RNA-seq data from 30 prenatal cortex samples and 43 postnatal dorsolateral prefrontal cortex samples in humans (Table S12). The RNA-seq data processing steps followed the same methodology as previously described.

### Quantification of the miRNA binding sites in the 3′ UTRs

Predicted human miRNA binding sites were obtained from TargetScan (Release 7.2) [70]. An miRNA binding site was assigned to the 3′ UTR when at least half of the miRNA binding peak was located within the 3′ UTR. The number of miRNA binding sites on 3′ UTRs was then quantified.

### Identification of orthologous ORF-disrupting PA sites in rhesus macaques and mice

The PA sites in rhesus macaque brains were identified using the datasets and pipeline we previously reported [61] with the incorporation of additional datasets from Warren and colleagues [71] (Table S11). For the identification of PA sites in mice, Iso-Seq data from mouse brain samples were downloaded from ENCODE and Leung et al. [72] (Table S11) and processed with the same pipeline. ORF-disrupting PA sites were then identified in both species based on the gene annotations for rhesus macaque (Ensembl v107) and mouse (GENCODE vM23). Finally, according to synteny data across humans, rhesus macaques, and mice, if the region orthologous to a human ORF-disrupting PA site was located within 30 bp of an ORF-disrupting PA site defined in rhesus macaques and/or mice, the sites were defined as orthologous ORF-disrupting PA sites.

### Identification of *ZNF271P* orthologs in out-group species

The regions orthologous to *ZNF271P* in chimpanzee (panTro3), rhesus macaque (rheMac3), mouse (mm10), dog (canFam3), and elephant (loxAfr3) were extracted from the synteny-based Gentree database [73]. To take advantage of the latest Ensembl gene annotations, the orthologous regions corresponding to genome assemblies used in Ensembl v106 were obtained using the University of California, Santa Cruz (UCSC) online LiftOver tool (http://genome.ucsc.edu/cgi-bin/hgLiftOver) with default parameters, if the conversions were available (Table S13). Otherwise, the sequence of the orthologous region was extracted, searched against the new assembly, and re-searched against the old assembly, and the reciprocal best alignment in the new assembly was then used.

In these regions orthologous to *ZNF271P* in other species, we further reviewed the gene model defined by Ensembl annotations (v106) and directly retrieved the gene models of *ZNF271P* orthologs in chimpanzee (ENSPTRG00000047230), rhesus macaque (ENSMMUG00000049532), mouse (ENSMUSG00000063281), and dog (ENSCAFG00845006933). For the ortholog of *ZNF271P* in elephants, the orthologous region was poorly annotated, and the gene model was reannotated using GeneWise (https://www.ebi.ac.uk/Tools/psa/genewise/) [74]. For *ZNF271P* orthologs in these species, the sequences for the longest transcripts and the corresponding protein sequences were then downloaded and subjected to multiple alignment using MUSCLE [75] and PRANK (v170417) [76], respectively. We also manually inspected the alignments according to the UCSC 100 vertebrates Multiz alignment track. Protein domains were predicted with InterPro (https://www.ebi.ac.uk/interpro/) [77]. To determine the percent match between the orthologous sequence and the reannotated human ZNF271P protein (based on chimpanzee), we utilized blastp (v2.5.0+) with default parameters [78].

### Ribo-seq data analyses

For ribosome-profiling sequencing (Ribo-seq) data processing, BAM files were obtained from https://www.ebi.ac.uk/arrayexpress/experiments/E-MTAB-7247/ [79], and only uniquely mapped reads were retained. Ribo-seq data from three replicates were merged for ORF prediction using RiboCode (v1.2.14) [80], in which the actively translated ORFs were predicted based on the 3-nucleotide (3-nt) periodicity of the footprints in ribosome profiling, with a threshold of adjusted *P* < 0.01.

### RNA-seq coverage of *ZNF271P* in the cross-species datasets

For the depiction of RNA-seq coverage of *ZNF271P* in developing brains of rhesus macaques and mice in Figure 2D, RNA-seq data were also acquired from the same study [64]. The rhesus macaque dataset included 12 prenatal brains ranging from embryonic day 93 to 130, and 25 postnatal samples spanned from postnatal day 0 to postnatal year 20–26 (Table S12). The mouse dataset included 59 prenatal brains ranging from embryonic day 10.5 to 18.5 and 39 postnatal samples spanned from postnatal day 0 to postnatal day 63 (Table S12).

### CRISPR/Cas9-mediated gene-editing of PA sites of *ZNF271P* in HEK293T cells

We first developed several CRISPR/Cas9 targeting constructs to knock out identified PA sites on *ZNF271P* in HEK293T cells (Figure S9). Briefly, the pmax-Cas9-GFP plasmid was built based on pmaxGFP (Catalog No. V4XP-3024, Lonza, Basel, Switzerland) by inserting a Cas9 sequence. Cas9 sequence and U6-gRNA sequence were obtained from PX458 (Catalog No. 48138, Addgene, Watertown, MA). pMini-gRNA was built based on puc19 (Catalog No. 3219, Takara, Kusatsu, Japan). CRISPOR (http://crispor.gi.ucsc.edu) was used to design the gRNAs and predict off-target loci.

To knock out the identified PA sites of *ZNF271P*, three gRNAs were designed to target the downstream of *ZNF271P* ORF to induce a 2850-bp deletion (gRNA-PAS-KO-1: 5′-AT TATTGATGAGTATGAAAA-3′(Forward strand), gRNA-PAS-KO-2: 5′-TCTTAGAAGCTCTGTACCAG-3′(Reversestrand), gRNA-PAS-KO-3: 5′-TTCAATAAATATTCAGTGTC-3′(Forward strand)). When performing knockout in HEK293T cells, a 1-μg pmax-Cas9-GFP plasmid and three 1-μg pmini-gRNA plasmids (gRNA-PAS-KO-1, gRNA-PAS-KO-2, and gRNA-PAS-KO-3) were transduced by Lipofectamine 3000 (Catalog No. L3000015, Invitrogen, Waltham, MA) according to the manufacturer’s protocol. After 40 h, Cas9-transduced GFP-expressing cells were sorted via FACS (FACSAria Fusion, BD, Franklin Lakes, NJ) and subsequently replanted. The expression levels of *ZNF271P* in the cultured HEK293T cells, both with and without gene editing, were quantified through RT-qPCR assays utilizing a pair of primers that target the ORF of *ZNF271P* (Forward:5′-AGTGCAGCAGAAGTTGTAGCC-3′,Reverse:5′-CTGGTACAGTGTCTCTGGTACTG-3′).

### Generation of *ZNF271P*-knockout and *ZNF271P*-knockout-rescue hESCs and human cortical organoids

The human embryonic stem cell line H9 (Catalog No. WA09, WiCell, Madison, WI) was maintained under feeder-free conditions with Essential 8 medium (E8, Catalog No. A1517001, Thermo Fisher Scientific, Waltham, MA), with routine mycoplasma contamination testing. For *ZNF271P* knockout construction, dual guide RNAs (gRNA-KO-1: 5′-CTTTTGCTACACTGATTACATGG-3′(Reverse strand),gRNA-KO-2: 5′-GGATCCTTCGATGTTTAATAAGG-3′(Reverse strand)) targeting the *ZNF271P* ORF were designed to delete a 41-bp fragment, generating a frameshift mutation (Figure 3A). Nucleofection was performed with Lonza 4D-Nucleofector. hESCs were dissociated into single cells by Accutase (Catalog No. A1110501, Gibco, Carlsbad, CA) and resuspended in P3 buffer (Catalog No. V4XP-3024, Lonza) at 1 × 10^6^ cells/100 μl, followed by co-electroporation with two 1.5-μg pMini-sgRNA constructs and 1.5-μg pmax-Cas9 plasmid using program CB-150. GFP-positive cells were selected via FACS after 40 h post-transfection and seeded at 300 cells/cm^2^. Monoclonal colonies were selected and sequenced using genotype-specific primers (Forward: 5′-GGGGAGAAACCATATAAATGTGATGT-3′,Reverse: 5′-GAAGGTTTTGCTACATTGATCACAG-3′). Cas-OFFinder (http://www.rgenome.net/cas-offinder/) was used to predict potential off-target sites, and the top 5 hits for each sgRNA were amplified by flanking primers to generate 200–400-bp products (Table S10), followed by Sanger sequencing validation. To build *ZNF271P*-knockout-rescue hESC lines, TET-ON-controlled expression of *ZNF271P* was knocked into the *AAVS1* locus by CRISPR/Cas9. We reconstructed a donor vector based on pAAVS1-PDi-CRISPRn (Catalog No. 73500, Addgene) by inserting the ORF of *ZNF271P*. Then, 1.5 μg of donor plasmid and 1.5 μg of U6-sgRNA-pMAX-Cas9 plasmid were used for a single nucleofection using program CB-150. After 24 h, 200 ng/ml puromycin (Catalog No. A1113803, Gibco) was added to selected positive colonies. Individual colonies were then picked and genotyped with primers (Forwardprimer1: 5′-TAACGCTGCCGTCTCTCTCCTGAG-3′, Reverseprimer1: 5′-ACCGTGGGCTTGTACTCGGT-3′, Forward primer 2: 5′-ACGCACTGTGGGGTGGAGATATC-3′, Reverse primer2: 5′-CCCAAAAGGCAGCCTGGTAGACA-3′) to select positive colonies. Subsequently, cortical organoids were differentiated from hESCs following a previously published protocol [7,10].

### Immunofluorescence staining

Organoids were harvested and fixed by 4% (w/v) paraformaldehyde. After infiltrated by 30% (w/v) sucrose solution, organoids were embedded in O.C.T. (Catalog No. 4583, Sakura, Torrance, CA) and cryosectioned into 20 μm slices. Cryosections were blocked by a blocking buffer containing 1× PBS, 5% (w/v) bovine serum albumin (BSA), and 3% (v/v) Triton X-100 at room temperature for 1 h. The samples were incubated overnight at 4°C with primary antibodies diluted by PBS solution containing 1% (w/v) BSA and 1% (v/v) Triton X-100 [antibodies: SOX2 (1:400; Catalog No. AF2018, R&D Systems, Minneapolis, MN), NeuN (1:200; Catalog No. ab177484, Abcam, Cambridge, UK), OCT-3/4 (1:500; Catalog No. 611203, BD), NANOG (1:200; Catalog No. ab80892, Abcam), phospho-Histone H3 (1:1000; Catalog No. 9701, CST, Danvers, MA), and mCherry (1:1000; Catalog No. PA5-34974, Thermo Fisher Scientific)]. The samples were treated with secondary antibodies and Hoechst 33342 (Catalog No. 62249, Thermo Fisher Scientific) for 2 h at room temperature and followed by mounting on glass slides. Image acquisition was carried out using an LSM880 confocal microscope (ZEISS, Baden-Württemberg, Germany), followed by digital processing with ZEN 2012 software suite and ImageJ analysis toolkit.

Statistical analyses were conducted using GraphPad Prism 6. Data with error bars were displayed as the mean ± SEM for all experiments. The unpaired two-tailed Student’s *t*-test was used to determine the statistical significance of differences between the two groups. *P* < 0.05 was considered to indicate a significant difference. ***, *P* < 0.001; **, *P* < 0.01; *, *P* < 0.05; ns, not significant.

### Single-cell preparations and scRNA-seq

Human cortical organoids grown for 60 days were washed twice with PBS and dissociated with Accutase. Each group contained over 10 organoids. Single-cell suspensions were subjected to cell sorting (FACSAria Fusion, BD). Finally, approximately 10,000 single cells were loaded onto Chromium chips to prepare the Chromium Single Cell 3′ library (v3; Catalog No. PN-1000268, 10X Genomics, Pleasanton, CA) according to the recommendations from the manufacturer.

The raw base calling files in bcl format were demultiplexed to FASTQ files with Cell Ranger (v7.1.0) [81], and an average of 700 million paired-end reads were generated for each sample. Raw reads were then aligned to the human reference genome (GRCh38/hg38) and Ensembl gene models (v107) using cellranger counts. The sparse gene count matrices were then used as the input to Seurat (v4.0.5) [82] for the downstream analyses. Stringent quality control was performed, with the removal of cells with fewer than 500 detected genes or more than 6500 detected genes and cells with a high percentage of mitochondrial genes (> 5%).

We then downsampled the number of cells in each sample to control for bias due to variations in cell number and obtained 13,720 cells (6860 in each sample) for further analyses. We also performed integration analyses to reduce batch effects in comparisons of different samples, in which the molecular count data in each sample were normalized using SCTransform based on regularized negative binomial regression, and 3000 variable features were used to find anchors for the integration. Based on the integrated data, principal component analysis was then conducted, and the top 30 principal components were used to identify the clusters (resolution 0.7) based on the Louvain algorithm implemented within Seurat (v4.0.5) [82], which were then visualized by t-distributed stochastic neighbor embedding (t-SNE) projection. Marker genes for each cell cluster were then identified using FindConservedMarkers in Seurat (v4.0.5, min.pct = 0.25) [82]. Cells unable to be classified were removed from the subsequent comparative analyses of cell composition. Differentially expressed genes between NPCs of organoids grown from *ZNF271P*-KO and *ZNF271P*-WT cells were identified using FindMarkers in Seurat (v4.0.5) (absolute value of average log_2_ fold change ≥ 1 and adjusted *P* < 0.05).

### Pseudotime analysis using Monocle

The differentiation trajectory of D60 organoids was reconstructed using R package Monocle (v2.28.0) through the following workflow: unique molecular identifier count data were modeled under a negative binomial distribution framework; trajectory-ordering genes were identified using dispersion-based selection criteria (mean expression ≥ 0.5 and empirical dispersion value ≥ 1); dimensionality reduction was performed using discriminative dimensionality reduction with trees method to project cells into two-dimensional space, with subsequent trajectory visualization through the plot_cell_trajectory function. For comparative analysis of differentiation progression, the pseudotime score was evenly divided into 10 intervals, and then the proportion of cells at the corresponding pseudotime interval was calculated.

### Transfection of HEK293T cells

HEK293T cells were cultured in Dulbecco’s modified Eagle’s medium (DMEM; Catalog No. 11965092, Life Technologies, Gaithersburg, MD) with 10% fetal bovine serum (Catalog No. 10091148, Gibco) and 0.1% penicillin/streptomycin (Catalog No. 15140122, Gibco). The ORF sequence of *ZNF271P* was cloned into pCDH-CAG-MCS-IRES-mCherry. HEK293T cells were transfected with plasmids (1 µg per 10^6^ cells) using GenEscort (Catalog No. WIS 2600, Wisegen, Nanjing, China) following the manufacturer’s instructions. After 48 h, cells were fixed and then subjected to immunofluorescence staining as described above.

## Supplementary Material

qzaf125_Supplementary_Data

## Data Availability

The single-cell transcriptome data generated in this study have been deposited in the Genome Sequence Archive for Human [83] at the NGDC, CNCB (GSA-Human: HRA010613), which is publicly accessible at https://ngdc.cncb.ac.cn/gsa-human/.

## Code availability

The code has been submitted to BioCode at the National Genomics Data Center (NGDC), China National Center for Bioinformation (CNCB) (BioCode: BT007913), which is publicly accessible at https://ngdc.cncb.ac.cn/biocode/tools/BT007913.

## CrediT author statement

**Ting Li:** Investigation, Formal analysis, Data curation, Writing – original draft. **Fan Mo:** Investigation, Validation, Writing – original draft. **Jianhuan Qi:** Investigation. **Chunqiong Li:** Investigation. **Xiangshang Li:** Investigation. **Jie Zhang:** Investigation. **Yingfei Lu:** Investigation. **Chao Yao:** Investigation. **Li Zhang:** Resources. **Baoyang Hu:** Resources. **Chuan-Yun Li:** Conceptualization, Supervision, Writing – review & editing, Funding acquisition. **Ni A. An:** Conceptualization, Supervision, Writing – original draft, Writing – review & editing, Funding acquisition. All authors have read and approved the final manuscript.
