# Supplementary material for: A Human-specific Protein Regulated by Alternative Polyadenylation Shapes Uniqueness of Human Brain Development
Source: Genomics Proteomics Bioinformatics. 2025 Dec 13;23(6):qzaf125. doi: 10.1093/gpbjnl/qzaf125 (PMC13197124; doi:10.1093/gpbjnl/qzaf125)
Supplement: qzaf125_Supplementary_Data [file qzaf125_supplementary_data.zip › Supplementary material captions 120625.docx]

## Supplementary material

**Figure S1** **Motif frequencies, nucleotide sequence compositions at the flanking regions, and the genomic distribution of PA sites**

**A.** Frequencies of hexamer motifs for sequences 50 bp upstream of the candidate PA sites identified in human brains. **B.** Nucleotide sequence compositions surrounding the candidate PA sites identified in human brains. **C.** Pie plot depicting the genomic locations of 65,994 identified PA sites. **D.** Pie plot depicting the genomic locations of 3717 ORF-disrupting PA sites. bp, base pair.

**Figure S2** **Distribution of ORF_PAU and gene expression levels between prenatal and postnatal brain samples**

**A.** Volcano plot showing the distribution of genes with differential ORF_PAU between the prenatal and postnatal stages. The blue dots denote shortening genes, the red dots denote lengthening genes, and the gray dots denote genes with no significant difference in ORF_PAU. **B.** Volcano plot showing the distribution of genes with upregulated and downregulated expression levels between prenatal and postnatal brains. The blue dots denote downregulated genes, the red dots denote upregulated genes, and the gray dots denote the genes not differentially expressed.

**Figure S3** **Comparison of gene expression levels and 3**′ **UTR lengths between ORF_PAU^+^Expr^+^ and ORF_PAU^+^Expr^−^ genes**

**A.** Comparison of gene expression levels between ORF_PAU^+^Expr^+^ and ORF_PAU^+^Expr^−^ genes. **B.** Comparison of 3′ UTR lengths between ORF_PAU^+^Expr^+^ and ORF_PAU^+^Expr^−^ genes. Wilcoxon test. ns, not significant. kb, kilobase pair.

**Figure S4 Heatmap of PA site usage of ORF_PAU^+^Expr^−^ genes across humans, rhesus macaques, and mice (shuffled datasets)**

The “PA site type” color bar indicates the type of PA sites: ORF-disrupting (orange) and ORF-containing (blue). The “Conservation” color bar indicates the conservation of the individual PA sites: Human_specific (green): detected exclusively in humans; HR_shared (light yellow): shared between humans and rhesus macaques; HM_shared (medium yellow): shared between humans and mice; HRM_shared (dark yellow): shared between humans, rhesus macaques, and mice. The color gradient in the heatmap represents PA site usage, with darker shades indicating higher usage. PA sites that could not be converted to rhesus macaques and/or mice are shown in gray for the respective species. Four ORF-disrupting PA sites on *ZNF271P* are labeled.

**Figure S5 Multiple sequence alignment of ZNF271P protein sequences**

Multiple sequence alignment of the sequences of C2H2 zinc finger contact residues of ZNF271P protein in six species, with the same alignments of nine C2H2 zinc finger contact residues folded. Percentages of the orthologous proteins in other species matching the human ZNF271P protein are shown on the right. MYA, a million years ago.

**Figure S6 Human-specific mutations in *ZNF271P***

The 4-bp deletion disrupting the ancestral ORF and a 1-bp insertion turning the out-of-frame ORF into an in-frame ORF relative to the ancestral protein are highlighted in red boxes. The UCSC 100 vertebrates Multiz alignment track was used to generate multiple sequence alignments with the following genome assemblies: human (hg38), chimpanzee (panTro4), rhesus macaque (rheMac3), mouse (mm10), dog (canFam3) and elephant (loxAfr3). UCSC, University of California Santa Cruz; PTC, premature stop codon.

**Figure S7 Boxplot showing the expression of ORF-containing isoforms of *ZNF271P* in prenatal or postnatal human brains**

Wilcoxon test, ****, *P* value ≤ 0.0001.

**Figure S8** **The ORF_PAU of *ZNF271P* in the prenatal and postnatal human brains from an independent dataset, corresponding to Figure 2D**

**A.** Normalized coverage of the RNA-seq reads of prenatal (*n* = 30) or postnatal (*n* = 43) human brains. The transcripts with all ORF-disrupting PA sites and the first ORF-containing PA sites were aligned and shown below the coverage tracks. The constitutive exon regions and alternative exon regions are indicated by the red and blue shaded areas. **B.** Boxplot showing ORF_PAU in prenatal or postnatal human brains. Wilcoxon test, ***, *P* value ≤ 0.001.

**Figure S9** **CRISPR/Cas9-mediated gene-editing of PA sites of *ZNF271P***

**A.** Overview of CRISPR/Cas9-mediated gene-editing in HEK293T cells, in which the gRNAs were designed to target the ORF-containing PA sites (PAS-KO) of *ZNF271P*. **B.** Relative expression levels of *ZNF271P* ORF quantified by RT-qPCR (*n* = 4 per group). Student’s *t*-test. **, *P* value ≤ 0.01. PAS, PA site.

**Figure S10** **Relative position of *ZNF271P* PA sites and the binding site peaks of *ELAV1* and *ELAV3* in the UCSC genome browser**

The blue panel indicates the binding site peaks of *ELAV1* and *ELAV3*. The orange panel denotes the PA sites of *ZNF271P*. The pink vertical bars represent the overlap between the PA sites and the CLIP-identified binding site peaks. CLIP, cross-linking and immunoprecipitation.

**Figure S11** ***ZNF271P* knockout in hESCs**

**A.** The expression of ORF-containing isoforms of *ZNF271P* in the human brain. **B.** The expression of ORF-containing isoforms of *ZNF271P* in cortical organoids. **C.** The design of the CRISPR/Cas9 assay to knock out *ZNF271P* with gRNA is shown (upper panel), and the knockout was further verified by Sanger sequencing (lower panel). **D.** PCR validation of the knockout assay. WT, wild type; Homozygous, the deletion of the target region of *ZNF271P* was present on both chromosomes.

**Figure S12** **Effects of *ZNF271P* knockout on hESC pluripotency**

**A.** Upper panel, immunofluorescence staining of pluripotency markers NANOG and OCT4 in wild-type hESCs (WT) and hESCs with *ZNF271P* knockout (KO). Lower panel, quantification of immunofluorescence staining. **B.** Upper panel, immunofluorescence staining of pluripotency marker SOX2 and proliferation marker PH3 in wild-type hESCs (WT) and hESCs with *ZNF271P* knockout (KO). Lower panel, quantification of immunofluorescence staining. *n* = 5. ns, not significant. Scale bars, 100 μm.

**Figure S13 Reconstruction of differentiation trajectory for organoids grown for 60 days**

**A.** Reconstructed differentiation trajectory of organoids grown for 60 days, with pseudotime scores represented by a gradient from dark blue (early stage) to light blue (late stage) (left panel) and cell types annotated (right panel). The black arrow indicates the direction of the developmental trajectory. **B.** Proportions of cells of organoids grown for 60 days from wild-type hESCs (D60_WT) and *ZNF271P*-knockout hESCs (D60_KO) across differentiation pseudotime.

**Figure S14 The ORF_PAU distribution of 1399 genes**

**Figure S15** **Investigation of batch effects in the identification of the developmental-related ORF-disrupting APA events**

**A.** PCA plots illustrating the removal of gender batch effects using ComBat. Left: before removing batch effects; Right: after removing batch effects. **B.** Normalized ORF_PAU of shortening and lengthening genes at subdivided time points of prenatal and postnatal stages. Different subdivided stages were clustered, with a clustering branch on the top of the heatmap, in which ORF_PAU was normalized by developmental stages. Embryonic: 4–7 weeks; Early fetal: 8–12 weeks; Early mid-fetal: 13,16,18 weeks; Late mid-fetal: 19 weeks. PCA, principal component analysis.

**Figure S16** **Subcellular localization of *ZNF271P***

**A.** Overview of the design of transfection experiments. **B.** Immunostaining of HEK293T cells transfected with CAG-mCherry (CTRL) or CAG-mcherry-271CDS (271) vectors. Scale bars, 10 μm.

**Figure S17 Phenotype of *ZNF271P*-KO organoids rescued by the expression of *ZNF271P* ORF**

**A.** Overview of the design of rescue experiments. **B.** Brightfield images showing the size of the representative organoids grown from *ZNF271P*-KO (KO) or rescue (RESCUE) hESCs at protocol day 60. Scale bars: 500 μm. **C.** Quantification of the average area of organoids grown from *ZNF271P*-KO (KO) and rescue (RESCUE) hESCs at protocol day 60. *n* = 20. **D.** Immunofluorescence staining of SOX2 (green) and NEUN (red) in Hoechst-stained (blue) organoids grown for 60 days generated from *ZNF271P*-KO (KO) and rescue (RESCUE) hESCs. Scale bars, 100 μm. **E.** Quantification of immunofluorescence staining. *n* = 4 organoids. *, *P* value ≤ 0.05; **, *P* value ≤ 0.01. **F.** Quantifications of *ZNF271P* expression in organoids grown from wild-type (WT), *ZNF271P*-KO (KO), and rescue (RESCUE) hESCs.

**Figure S18** **Expression of ORF-containing isoforms in six organs (*n* = 18 per group)**

**Table S1 65,994 PA sites in 19,045 genes identified by Iso-Seq data**

**Table S2 17,686 PA sites situated within the 5815 genes with a single open reading frame**

**Table S3 Full list of 1399 genes and 216 genes generated in this study**

**Table S4 The ORF_PAU of 136 orthologous genes across three species**

**Table S5 332 PA sites disrupting ORFs of 136 human genes and their orthologous ORF-disrupting sites in macaques and mice**

**Table S6 Metadata of human samples used in gnomAD v4**

**Table S7 PA sites on *ZNF271P* and corresponding PA site motifs**

**Table S8 77 RBPs interacting with *ZNF271P* supported by large-scale CLIP-seq data from starBase v2.0**

**Table S9 Full list of differentially expressed genes and their functional enrichment between NPCs of organoids grown from *ZNF271P*-KO and *ZNF271P*-WT cells**

**Table S10 Primers for off-target analysis**

**Table S11 Metadata of public Iso-Seq datasets used in this study**

**Table S12 Metadata of public RNA-seq datasets used in this study**

**Table S13 The gene models of *ZNF271P* orthologs annotated by Ensembl 106**
