# Supplementary material for: A Human-specific Protein Regulated by Alternative Polyadenylation Shapes Uniqueness of Human Brain Development
Source: Genomics Proteomics Bioinformatics. 2025 Dec 13;23(6):qzaf125. doi: 10.1093/gpbjnl/qzaf125 (PMC13197124; doi:10.1093/gpbjnl/qzaf125)
Supplement: qzaf125_Supplementary_Data [file qzaf125_supplementary_data.zip › Table S6.docx]

| **Table S6 Metadata of human samples used in gnomAD v4** | | |
| --- | --- | --- |
| **Feature type** | **Feature** | **Sample count** |
| Gender | Females | 400,897 |
|  | Males | 406,265 |
| Race | Admixed American | 30,019 |
|  | African/African American | 37,545 |
|  | Ashkenazi Jewish | 14,804 |
|  | East Asian | 22,448 |
|  | European | 622,057 |
|  | Middle Eastern | 3031 |
|  | Remaining | 31,712 |
|  | South Asian | 45,546 |
| Age | < 30 | 9652 |
|  | 30-35 | 4669 |
|  | 35-40 | 5207 |
|  | 40-45 | 48,016 |
|  | 45-50 | 63,811 |
|  | 50-55 | 75,306 |
|  | 55-60 | 86,629 |
|  | 60-65 | 111,640 |
|  | 65-70 | 86,238 |
|  | 70-75 | 8247 |
|  | 75-80 | 4016 |
|  | > 80 | 2388 |
|  | Not available | 301,343 |
