# Supplementary material for: A Human-specific Protein Regulated by Alternative Polyadenylation Shapes Uniqueness of Human Brain Development
Source: Genomics Proteomics Bioinformatics. 2025 Dec 13;23(6):qzaf125. doi: 10.1093/gpbjnl/qzaf125 (PMC13197124; doi:10.1093/gpbjnl/qzaf125)
Supplement: qzaf125_Supplementary_Data [file qzaf125_supplementary_data.zip › Table S10.docx]

| **Table S10 Primers for off-target analysis** | |
| --- | --- |
| **Target** | **Sequence** |
| gRNA1-OF1-F | GTGGAGAGGTGGAGGGTTATTG |
| gRNA1-OF1-R | CCAGAGCAGACTGTGTTATGTTTA |
| gRNA1-OF2-F | CTTCCCTTTCTTCACCCACCT |
| gRNA1-OF2-R | CCCAAAGGGCAGTGATAAACC |
| gRNA1-OF3-F | TGTGAACTGCTCTCAGCTCCT |
| gRNA1-OF3-R | AGATTTCCCTCCTTGCCATCC |
| gRNA1-OF4-F | AAGAGTCCCTTTGAGGAAATCAG |
| gRNA1-OF4-R | TGTTTCACTTTTGTTTCCTGTTGG |
| gRNA1-OF5-F | TAAGCATGTGCCCTTGTGGT |
| gRNA1-OF5-R | TGTGTACTGGTCCATGAAAGGT |
| gRNA2-OF1-F | GTCGAAATTCATTGCTTGTTGAGC |
| gRNA2-OF1-R | ACTAAAGGCTTTTCCACATTCCTC |
| gRNA2-OF2-F | TGTGGGAAAAGTTTTAGTCGCA |
| gRNA2-OF2-R | GGTGTGATTTCCAACTGAAGGC |
| gRNA2-OF3-F | GAAAGGCAATTGTGGCAGACAG |
| gRNA2-OF3-R | CTCCATCTTCAGAGCCAGTGAC |
| gRNA2-OF4-F | TGTCATCAGTGGAGCACAAAT |
| gRNA2-OF4-R | TGGCAATTAATGGGACCTGC |
| gRNA2-OF5-F | AGCGACCAACTGATCAATCAAAAA |
| gRNA2-OF5-R | TTCACCTTACAGATTCATCAACAAA |
